# A method for automated pathogenic content estimation with application to rheumatoid arthritis

**DOI:** 10.1186/s12918-016-0344-6

**Published:** 2016-11-15

**Authors:** Xiaoyuan Zhou, Christine Nardini

**Affiliations:** 1 Group of Clinical Genomic Networks, Key Laboratory of Computational Biology, CAS-MPG Partner Institute for Computational Biology, Shanghai Institutes for Biological Sciences, Shanghai, People’s Republic of China; 2 University of Chinese Academy of Sciences, Beijing, People’s Republic of China; 3 Personalgenomics, Verona, Italy

**Keywords:** Microbiome, Pathogens, Rheumatoid arthritis

## Abstract

**Background:**

Sequencing technologies applied to mammals’ microbiomes have revolutionized our understanding of health and disease. Hence, to assess diseases’ progression as well as therapies longterm effects, the impact of maladies and drugs on the gut-intestinal (GI) microbiome has to be evaluated. Typical metagenomic analyses are run to associate to a condition (disease, therapy, diet) a pool of bacteria, whose eubiotic/dysbiotic potential is assessed either by α-diversity, a measure of the varieties populating the microbiome, or by Firmicutes to Bacteroides ratio, associated to systemic inflammation, and finally by manual and direct inspection of bacteria’s biological functions, when known. These approaches lead to results sometimes difficult to interpret in terms of the evolution towards a specific microbial composition, harmed by large areas of unknown.

**Results:**

We propose to additionally evaluate a microbiome based on its global composition, by automatic annotation of pathogenic genera and statistical assessment of the net varied frequency of harmless versus harmful organisms. This application is intuitive, quantitative and computationally efficient and designed to cope with the currently incomplete species’ functional knowledge. Our results, applied to human GI-microbiome data exemplify how this layer of information provides additional insights into treatments’ impact on the GI microbiome, allowing to characterize a more physiologic effects of Prednisone versus Methotrexate, two treatments for rheumatoid arthritis (RA) a complex autoimmune systemic disease.

**Conclusions:**

Our quantitative analysis integrates with previous approaches offering an additional systemic level of interpretation here applied, for its potential to translate into clinically relevant information, to the therapies for RA.

**Electronic supplementary material:**

The online version of this article (doi:10.1186/s12918-016-0344-6) contains supplementary material, which is available to authorized users.

## Background

With the development of high-throughput technologies, large amounts of metagenomic data have been produced, especially with the sequencing of the 16S ribosomal RNA gene, used as proxy for taxa abundances in a microbial community. This has demonstrated how the gut intestinal (GI) microbes respond and adapt to different situations [1], how alterations of the microbial community impact on the development and functioning of the immune and metabolic systems [2], and, globally, how divergences from homeostasis (eubiosis) in this district are predictive of diseases (dysbiosis). Typical approaches to analyze these data consist of the evaluation of the α-diversity of Operational Taxonomic Units (OTUs, computational proxies for species) within each sample to understand the microbial population structure using Shannon [3] and Simpson [4] indexes. This is based on the observation that more variability offers a larger spectrum of microbial molecular functions and hence of responses to environmental variations [5], and, reversely, this criterion relies on the observed limited α-diversity in inflammatory bowel disease [6] and obesity [7].

Along the same line, evaluation of the imbalance in the physiologic abundances of Bacteroides and Firmicutes is observed to be a measure of the inflammatory state of the system and a proxy for dysbiosis due to the relative increase of facultative anaerobic microbes able to exploit byproducts of the host inflammatory processes [8].

From a different perspective, differential analyses compute microbial variations, and highlights OTUs whose abundance are significantly changed between two conditions, followed by annotation of OTUs to taxa and manual search of known organisms whose functions within the host environment help to shed light, for example, on the mechanisms that trigger or sustain a disease.

Worldwide, large efforts are ongoing to complete the taxonomy of mammalians’ microbes, with a particular focus on their effects on health and disease (Human Microbiome Project, HMP) in synergy with metatranscriptomics and metaproteomics analyses to elucidate functional information [9]. Nevertheless, little is still known to date. As a result, despite the possibility to screen GI microbiomes at relatively low costs and with minimal invasiveness, it remains difficult to gain global understanding on the beneficial or deleterious effect of a condition, limited by the known bacteria (functions), thus leaving unaddressed, for example, the impact a novel therapy on the GI tract and, in the long run, on the immune and metabolic systems.

While awaiting for a (more) complete characterization of bacteria in the human GI microbiome, we propose to add a layer of interpretation by quantification of the varied composition of pathogens, with respect to a baseline, in statistical terms. This represents an informed base to further screen specific strains.

In fact, microbiology has cumulated, on harmful bacteria, a remarkable amount of information. From the well and long known *Mycobacterium tuberculosis* [10], more recent findings have shown how previously unsuspected noncommunicable diseases are also affected by bacterial alterations leading to the characterization of *Porphyromonas gingivalis* [11] in the mouth microbiome and *Prevotella copri* [12] in the GI microbiome as drivers of RA and to *Lactobacilli*-rich food conversely reported to improve RA symptoms [13].

As a result, it is possible to define bacteria as *harmful* when explicitly associated to a disease, or *harmless* (rather than beneficial, in a conservative perspective) otherwise. The collection of such information is not yet centralized, and we here offer a first curated database of this type of classification (part of the eudysbiome package, also added as Additional file 1: Table S1 for convenience).

This approach overcomes two current lacks: on one side, efficient and automated usability of the pathogenic potential information; and on the other side, a genera annotation strategy capable to fill the paucity of information available at the OTU level. Namely, we overcome these issues by: (i) centralizing available pathogenic annotation resources; (ii) devising a pathogenic genera definition, both implemented in a statistical pipeline available as Bioconductor package, offering tabular and graphical output.

Two words of cautions must be put forward for the usage of this approach. First, to offer the most detailed annotation we rely on OTUs/species (see Methods), that however imply a number of unknown/unannotated elements discarded from further analyses to avoid bias in the results. Second, the abundance of pathogens must be put into context, for example, healthy and long-lived hunter-gatherer populations are characterized by GI microbiomes with higher α-diversities than urban populations [14], including in this diversity numerous pathogens; however, when comparing the effects of treatments on a clinically uniform set of patients, the increased abundance of pathogens represents an added risk of comorbidity in individuals with already debilitated general health conditions. It is recommended, as in any *omic* analysis, to further manually investigate such global harmless/harmful trends by manual investigation of the emerging strains (as it is done for example in transcriptomics with the manual inspection of the genes identified in a statistically significant Gene Ontology biological function).

Globally, this approach should be considered as integrative and complementary to the existing ones to shed additional light on the effects of maladies, treatments and other external input on the host-microbiome supra-organism. To present the usability and informativeness of this approach, we apply it to the analysis of the GI microbiome of patients affected by rheumatoid arthritis (RA), a model for chronic, inflammatory and autoimmune diseases, spreading at very fast pace, and whose microbial composition is being continuously unveiled. For its incidence (1 % worldwide) and its exemplar characteristics (model disease) our results represents not only an important example of application but also meaningful results *per se*.

## Implementation

### Reference database

**Fig. 1:**
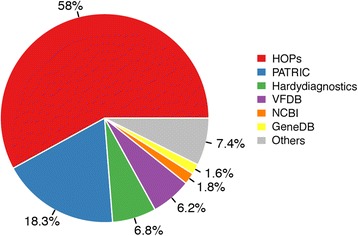
Statistics of pathogenic species in reference databases

The human bacteria pathogens were integrated into a Genus-Species table by collecting lists of microbes annotated as pathogens based on metagenomes information (references 1–3); virulence factors used to assess infections (reference 4); clinical studies to be frequently found in diseases (references 5–6) as summarized in Fig. 1:

1. National Center for Biotechnology Information (NCBI) Pathogen Detection system (http://www.ncbi.nlm.nih.gov/pathogens/), using information on human pathogens (not foodborne pathogens) of “Acinetobacter” and “Klebsiella”;
2. Genome Database of Pathogens (GeneDB, [15]) for prokaryotic and eukaryotic pathogens and closely related organisms, collected via downloading the bacteria information in a “protein-coding” Gene Type giving rise to 12 pathogenic genera;
3. Pathosystems Resources Integration Center (PATRIC, [16]), a bacterial information system with 2365 bacteria genomes hosted by humans and involved in diseases;
4. Virulence Factor Database (VFDB, [17]), an integrated and comprehensive online resource for virulence factors of 30 pathogenic genera and related species;
5. Human Opportunistic Pathogens (HOPs) library, collected by the Gifu University, Genetic Information Genetic Resource Center of Human Pathogens (http://gtc.jpn.com/?p=1);
6. “Indigenous and pathogenic microorganisms by human body site”, by the Hardy Diagnostics company (https://catalog.hardydiagnostics.com/cp_prod/Content/hugo/IndigPathogOrganisms.htm) with two attributes: *frequency* (expected in a clinical specimen, from 1 to 3) and *pathogenicity* (expected when the organism is present, ≧2).

Additional missing species were searched in Pubmed with query terms < *species name*, human, pathogen>, manual screening of the resulting literature, and finally update into the above Genus-Species table.

### *eudysbiome* R package

The package eudysbiome is developed in the statistical computing environment R and is released under the GNU General Public License within Bioconductor [18]. It performs the analysis including species-level classifications of unknown 16S rRNA sequences, genus annotation as *harmful* or *harmless* based on the described pathogenic Genus-Species table above, and tests the association between microbial variations and a given condition.

The package takes as input a list of differential microbes abundances’ (reads) variation (Δg = g1 – g2) defined as the difference between a genus’ abundance in condition1 (g1) and at the baseline condition2 (g2). The calculation of Δg is left to the users, given the different types of normalizations and considerations to be done on a case by case basis. We here recommend to use limma [19] for good performance on small sample data, and tools such as metagenomeSeq [20], LefSe [21], metastats [22] for more general cases.

As a genus can collect under its name both harmful and harmless species, the proper annotation of a genus as *harmless* or *harmful* can benefit from the investigation of the species actually present in each dataset, so that, if a genus, including by definition also harmful species, does not include them in a specific sample, the genus can be annotated as *harmless*. By the same token, if none of this genus’ species actually appears in the data under study, the genus is discarded from the analysis for lack of (annotation on the) species, leading to the impossibility to annotate the genus as *harmful*/*harmless*. eudysbiome allows this (optional) more careful species classification and hence annotation, even in the case where the input data is given in the form of differential genera by directly calling the Mothur [23] command “classify.seqs” and mapping the unknown 16S rRNA sequences to a well-curated representative dataset of 16S rRNA reference sequences by Wang’s naïve Bayesian classifier, recognized as an efficient method and accurate classifier [24, 25]. To guarantee a fast species-level classification and minimize the needed computational resources, the package rely on the latest QIIME [26] released SILVA [27] (16S/18S, SSU119, https://www.arb-silva.de/no_cache/download/archive/qiime/) representative set created by clustering at 97 % sequence identity. After the annotated Δgs are made available, the package permits to group frequencies |Δg| into ∑|Δg| as increase of harmless bacteria abundances plus decrease (absolute value) of harmful bacteria abundances for the eubiotic contributions and viceversa for the dysbiotic. This is visually represented in a Cartesian plane with *harmful*/*harmless* microbes on the x-axis and ∑|Δg| on the y-axis, and summarized in a Condition × Impact table, both outputs of the package. The package further evaluates statistically the abundance of harmless/harmful variation’s impact of a given condition on the microbiome, in comparison to the microbiome of the reference condition. To elaborate the significance of the association between conditions and eubiotic/dysbiotic impacts, Fisher's exact test [28] is used on the frequency counts for testing the null hypothesis that conditions are equally likely to lead to a mostly harmless-composed microbiome when compared to the control (two-sided) or that one condition is more likely to be associated to a mostly harmless microbiomes than the other (one-sided Fisher).

### Application to rheumatoid arthritis (RA)

16S rRNA genes from human samples collected in [12] represent the GI microbiomes of RA patients, either newly diagnosed (new onset RA, NORA) or chronically affected (Chronic RA, CRA), as well as psoriatic arthritis patients (PsA) treated with methotrexate (MTX), prednisone, opioids and, optional for all treatments, nonsteroidal anti-inflammatory drugs (NSAIDs). These data are analyzed, in the manuscript of origin, in search of disease-associated (NORA, CRA, PsA) variations of the GI microbiome in comparison to a healthy (HLT) baseline, independently of the therapy. Here, we deepened the investigation in search of RA treatment-associated GI variations. Irrespectively on the assumption of NSAIDs, samples were selected and re-grouped into five arms: 39 untreated new-onset rheumatoid arthritis (NORA), 11 untreated chronic rheumatoid arthritis (UCRA), 9 CRA samples treated with MTX (MTX), 3 CRA samples treated with prednisone (Prednisone) and 28 healthy controls (HLT). The only patient treated with opioids was removed from the analysis and so were the PsA patients. The representative sequences for each OTU and the OTUs abundance table with read counts down to the genus classification were downloaded from https://github.com/polyatail/scher_et_al_2013/tree/master/16S_Analysis.

### Microbial diversity and differential analysis

OTU-based diversity was evaluated on read counts by Shannon [3] and inverse Simpson index [4] calculated by the R Vegan package [29] and averaged among samples in each arm for comparisons. OTUs were grouped at the genus level before differential analysis and genera lacking of genus classifications were classified to their higher-order taxonomy. To minimize the noise associated to low abundance, reads with small within group variance, genera with null abundance in more than 1 sample or summed abundance among samples below 5, were filtered out. Abundances were further normalized with trimmed mean of M-values (TMM) and converted to log2-cpm (counts per million) by Voom in the edgeR package to make data suitable to linear regression in limma differential analysis. Significantly differential genera were selected by fold change (FC > 2) and p-value (*p* < 0.05), differential ones with higher-order classifications were removed from further analyses.

## Results and Discussion

The original analysis by Scher et al. [12] focuses on the GI variations from a healthy baseline (HLT) in association to a (stage of the) disease (NORA, CRA, PsA). As drug interventions strongly affect the immune response via the modulation (also) of the GI microbiome [30], we deepen the characterization of the GI microbiomes, disease-wise and explore additionally the effects of RA on the GI microbiome, therapy-wise (NORA, UCRA, MTX, Prednisone).

**Fig. 2:**
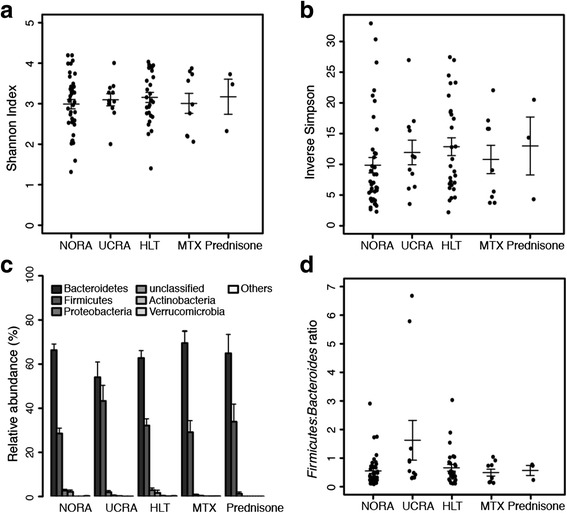
Microbial community structure in RA 16S rRNA-seq samples. **a**. Shannon index **b**. inverse Simpson index **c**. Phyla histogram **d**. Firmicutes to Bacteroides ratio. Data are presented as mean ± s.e.m. (standard error of mean)

By both measures of α-diversity (Fig. 2a-b), NORA appears to be the most severely affected by a reduced α-diversity, followed by UCRA and MTX, further followed by HLT and Prednisone. Comparable α-diversities in the two latter arms (HLT and Prednisone) suggest that Prednisone well controls the RA-associated dysbiosis allowing for a spectrum of species within the GI district that is broader than the one allowed by UCRA and MTX, and comparable to the physiological (HLT) α-diversity.

By the Firmicutes/Bacteroides criterion (Fig. 2c), the UCRA arm stands out with a ratio 2.4, 2.9, 3.3 and 2.8 folds higher than HLT, NORA, MTX and Prednisone, respectively (Fig. 2d), matching the well known inflammatory/dysbiotic state of UCRA patients. Globally we can conclude that the progression of the disease (NORA to CRA) is characterized by increasing diversity, where the increasing OTUs variety falls into the Firmicutes phylum (at the expenses of Bacteroides [8]).

It seems that once UCRA patients receive treatment, MTX lowers the diversity (Fig. 2a-b) and the inflammatory environment (Fig. 2c-d) bringing the system back to levels characteristic of the earlier stage of the disease (NORA), while Prednisone allows for a more physiological gain of diversity (Fig. 2a-b) and inflammatory environment (Fig. 2c-d), seemingly bringing the state of the GI closer to the HLT samples.

**Fig. 3:**
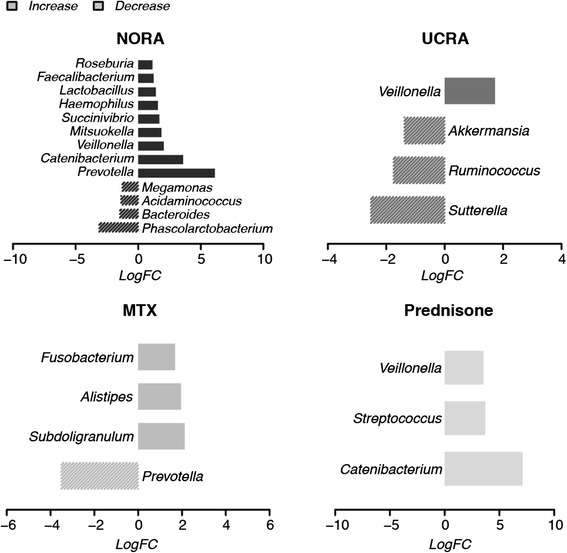
Variations of differential genera. Identified by limma (FC > 2, *p*-value < 0.05)

**Fig. 4:**
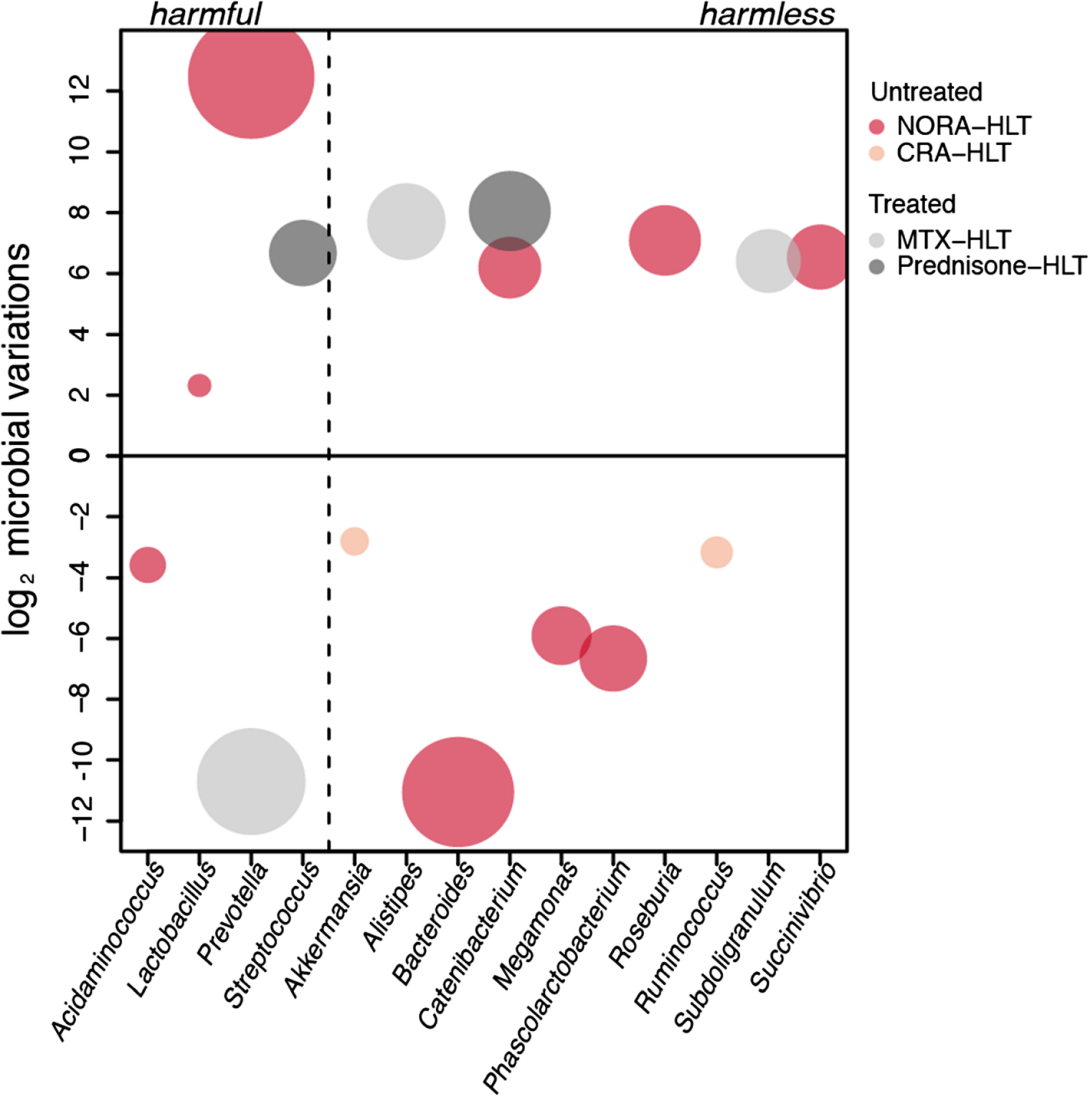
Cartesian plane of eubiotic/dysbiotic impacts. Harmful/harmless annotated genera (x-axis) and their abundance variations (Δg) among the compared condition (y-axis)

To gain further insight into these mechanisms, OTU representative sequences were classified into species by mapping to SILVA representative sequences at 97 % similarity with eudysbiome package (see elapsed time of taxonomic classification in Additional file 2: Table S2), building on further differential analysis (Fig. 3 and Additional file 3: Table S3) we additionally characterized the variations among these compositions by eudysbiome. Table 1b shows a striking and significantly different contribution of pathogens in the untreated versus treated arms that can be explored further in Fig. 4 that details the figures in Table 1a.

**Table 1:** Contingency and contingency tests with HLT baseline

a. Contingency ∑|Δg|	Eubiotic frequency		Dysbiotic frequency	
NORA-HLT	314		7977	
UCRA-HLT	0		16	
MTX-HLT	1965		0	
Prednisone-HLT	266		102	
b. Contingency test *p*-values	NORA	UCRA	MTX	Prednisone
NORA		0.54	1	1
UCRA	1		1	1
MTX	***0***	***3.95E-40***		1
Prednisone	***1.24E-251***	***2.99E-09***	1	

a. Condition-impact contingency table with cumulated frequencies accounted for harmless and pathogenic impacts (column) under the compared conditions (row). ∑|Δg| is the result of an increase of harmless bacteria abundances plus decrease (absolute value) of harmful bacteria abundances for eubiotic microbiomes and viceversa for dysbiotic. b. Contingency test assessing the hypothesis that the condition in the row is more associated to a more harmless composition than the condition in the column. One-sided Fisher’s exact test, *p* < 0.05, in bold)

In particular, we can see that the eubiotic trend in Prednisone is due to the sole contributions of increasing harmless genera (1^st^ quadrant in Fig. 4, Eubiotic frequency = 266 in Table 1a), limited by a dysbiotic contribution given by the increase of pathogens (2^nd^ quadrant in Fig. 4 and Dysbiotic frequency = 102 in Table 1a). Differently, MTX presents only eubiotic variations (Dysbiotic frequency = 0 in Table 1a), obtained by the two fold contribution of harmless genera increase (1^st^ quadrant) and pathogens’ decrease (3^rd^ quadrant, globally reaching the Eubiotic frequency = 1965 in Table 1a). This leads, remarkably, in the MTX samples to the reduction of the population of *Prevotella*, well known trigger of the disease [12], which remains conversely uncontrolled in Prednisone.

These results account for variations across a large number of species in the GI suggesting a systemic effect broader than the the host metabolism as anti-inflammatory action known for Prednisone [31] and the host anti-proliferative effect for MTX [32]. Indeed despite the well known limits of MTX and although its therapeutic activity is known to be associated to adverse effects also in the GI districts [33], not enough focus has been put yet on the broader impact of drugs on the patients as a whole, and only marginal attention is put to compensate such detrimental events with GI protective or boosting strategies [13, 34].

## Conclusions

In order to help elucidate the functionalities promoted or harmed in the GI district by diseases and other environmental triggers, we propose to integrate the study of the composition of the GI microbiome with an automated and statistical characterization of its pathogenic potential. Application of this approach should be done in synergy with current approaches like the study of α-diversity and the Firmicutes/Bacteroides ratio. In particular we present an application to rheumatoid arthritis, a model malady for all autoimmune diseases (including diabetes), whose etiology and control at the microbiome level represent a critical topic in clinical research and we show how the addition of the pathogenic information can help in differentiating the forces at work in the complex host-microbiome interaction system.

## Additional files

Additional file 1: Table S1. Human bacteria pathogens in a Genus-Species table collected from six public database and manual searching, this table is integral to the eudysbiome package, reported here for convenience. (XLSX 73 kb)
Additional file 2: Table S2. a. Running platform and b. elapsed time of species classification by applying eudysbiome package on RA data. (XLSX 35 kb)
Additional file 3: Table S3. Differential genera by comparison of NORA, UCRA, MTX, Prednisone with HLT arms. (XLSX 43 kb)

## Abbreviations

CRA: Chronic rheumatoid arthritis
GI: Gut intestinal
HLT: Healthy
MTX: Methotrexate
NORA: New onset rheumatoid arthritis
NSAIDs: Nonsteroidal anti-inflammatory drugs
OTU: Operational taxonomic unit
PsA: Psoriatic arthritis patients
RA: Rheumatoid arthritis
TMM: Trimmed mean of M-values
UCRA: Untreated CRA
